# Cellular and Molecular Mechanisms Underlying Altered Excitability of Cardiac Efferent Neurons in Cirrhotic Rats

**DOI:** 10.3390/biomedicines12081722

**Published:** 2024-08-01

**Authors:** Choong-Ku Lee, Huu Son Nguyen, Seong Jun Kang, Seong-Woo Jeong

**Affiliations:** Laboratory of Molecular Neurophysiology, Department of Physiology, Yonsei University Wonju College of Medicine, Wonju 26426, Republic of Korea; clee@mpinat.mpg.de (C.-K.L.); huuson2196@gmail.com (H.S.N.); dnagnpfgnpf@naver.com (S.J.K.)

**Keywords:** calcium channels, cardiac autonomic dysfunction, cirrhosis, heart-rate variability, intracardiac ganglia, potassium channels, stellate ganglia

## Abstract

Patients with cirrhosis often exhibit cardiac autonomic dysfunction (CAD), characterized by enhanced cardiac sympathetic activity and diminished cardiac vagal tone, leading to increased morbidity and mortality. This study delineates the cellular and molecular mechanisms associated with altered neuronal activities causing cirrhosis-induced CAD. Biliary and nonbiliary cirrhotic rats were produced by common bile duct ligation (CBDL) and intraperitoneal injections of thioacetamide (TAA), respectively. Three weeks after CBDL or TAA injection, the assessment of heart rate variability revealed autonomic imbalance in cirrhotic rats. We observed increased excitability in stellate ganglion (SG) neurons and decreased excitability in intracardiac ganglion (ICG) neurons in cirrhotic rats compared to sham-operated controls. Additionally, threshold, rheobase, and action potential duration exhibited opposite alterations in SG and ICG neurons, along with changes in afterhyperpolarization duration. A- and M-type K⁺ channels were significantly downregulated in SG neurons, while M-type K⁺ channels were upregulated, with downregulation of the N- and L-type Ca^2^⁺ channels in the ICG neurons of cirrhotic rats, both in transcript expression and functional activity. Collectively, these findings suggest that cirrhosis induces an imbalance between cardiac sympathetic and parasympathetic neuronal activities via the differential regulation of K^+^ and Ca^2+^ channels. Thus, cirrhosis-induced CAD may be associated with impaired autonomic efferent functions within the homeostatic reflex arc that regulates cardiac functions.

## 1. Introduction

Autonomic control of cardiovascular functions is essential for maintaining optimal blood flow to all organs in the body, meeting the metabolic demands under both normal and stressed conditions. This control is primarily achieved through reflexive regulation of the sympathetic and parasympathetic nervous systems. However, the cardiovascular homeostatic mechanism is frequently impaired in chronic liver diseases, regardless of the underlying cause [1,2,3,4]. In patients with cirrhosis, the prevalence and severity of cardiac autonomic dysfunction (CAD) increase with progressing hepatic dysfunction, elevating the risk of morbidity and mortality [2,5,6]. Cirrhosis is associated with portal hypertension, which leads to hyperdynamic circulation syndrome, characterized by increased cardiac output, decreased peripheral vascular resistance, and reduced circulating volume [7]. There is some evidence suggesting that CAD may contribute to the pathogenesis of these hemodynamic disturbances in cirrhotic patients [8,9].

The presence of CAD in cirrhosis is characterized by reduced baroreflex sensitivity (BRS) and heart rate variability (HRV). Typically, the diminished BRS observed in cirrhotic patients reflects decreased parasympathetic vagal activity, leading to a sympathovagal imbalance [3,5,10]. Elevated levels of circulating norepinephrine in these patients further suggest excessive sympathetic nervous system activity [11,12,13]. Power spectral analysis of HRV has demonstrated that both sympathetic and parasympathetic nervous systems are synchronously affected in cirrhosis [14,15], indicating autonomic imbalance characterized by sympathetic overactivity and reduced parasympathetic function.

Cirrhosis-induced CAD may result from functional alterations at various levels of the arterial baroreflex circuitry, which encompasses the afferent and efferent limbs as well as the central autonomic pathways. Previous research has shown that cirrhosis increases the expression of c-Fos, a marker of neuronal activation, in the nucleus of the solitary tract (NTS) and the rostral ventrolateral medulla (RVLM), potentially enhancing the sympathetic outflow to spinal preganglionic neurons [16]. At the peripheral level, our research has demonstrated that the cirrhosis-associated blunting of BRS can be attributed to the decreased excitability of A- and C-type aortic baroreceptor neurons [17]. A key ionic mechanism responsible for this decreased excitability involves the downregulation of voltage-gated sodium channels. In this study, we further explored whether the excitability of cardiac efferent neurons was similarly altered in both biliary and nonbiliary cirrhotic rats and examined the ionic mechanisms underlying the functional plasticity. We found that cirrhosis-induced CAD, confirmed by reduced HRV, was associated with the increased excitability of sympathetic stellate ganglion (SG) neurons and the decreased excitability of parasympathetic intracardiac ganglion (ICG) neurons. Notably, these changes in excitability stem from the downregulation of A- and M-type K^+^ channels in SG neurons and the upregulation of M-type K^+^ channels along with the downregulation of N- and L-type Ca^2+^ channels in ICG neurons.

## 2. Materials and Methods

### 2.1. Animals

Male Sprague-Dawley rats weighing 200–250 g were obtained from DBL Co. (Eum-seong-gun, Republic of Korea) and housed in individual cages under a 12:12 h light/dark cycle at a controlled temperature of 21–22 °C. They had ad libitum access to food and water. All animal care and experimental procedures were conducted in accordance with the National Institutes of Health’s Guidelines for the Care and Use of Experimental Animals and were approved by the Institutional Animal Care and Use Committee (IACUC) of Yonsei University Wonju College of Medicine (Approval No. YWC-120831-1).

### 2.2. Induction of Cirrhosis

**Figure 1:**
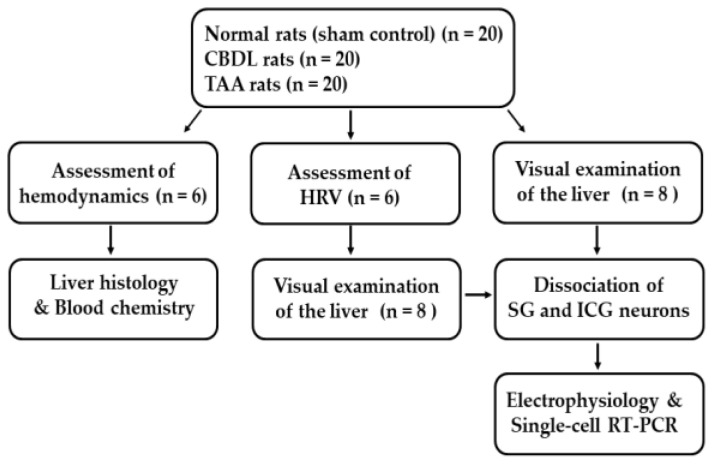
Design of the experiments. Rats were allocated into three groups: sham-control (n = 20), CBDL (n = 20), and TAA (n = 20). Body weight and hemodynamic parameters were measured in the control and cirrhotic rats three weeks post-sham-operation or post-CBDL, and six weeks post-saline or TAA injections. Subsequent to hemodynamic assessment, liver histology and blood analysis were conducted. HRV was evaluated in six rats per group via ECG recordings to confirm the development of CAD. Following ECG recording, liver tissues were visually inspected, and SG and ICG were harvested for the dissociation of individual neurons for electrophysiological measurements and single-cell RT-PCR. This protocol was also followed for rats in each group not utilized for hemodynamics and HRV assessments.

As illustrated in Figure 1, animals were randomly allocated into three groups: (i) the normal (sham-control) group (n = 20), (ii) CBDL group (n = 20), and (iii) TAA group (n = 20). Biliary and nonbiliary cirrhotic rats were generated using common bile duct ligation (CBDL) and intraperitoneal injection of thioacetamide (TAA) (Sigma-Aldrich, St. Louis, MO, USA), respectively, as previously described [17,18]. Briefly, rats were anesthetized with a ketamine-rompun mixture (50 mg/kg ip) following an overnight fast (12 h). For the CBDL procedure, a midline laparotomy was performed immediately below the xiphoid process; the common bile duct was doubly ligated with 4-0 silk and cut between the ligatures. Sham-operated (normal) rats underwent laparotomy without CBDL. To induce nonbiliary cirrhosis, rats received intraperitoneal injections of either saline or TAA (200 mg/kg) three times per week for six weeks [18,19]. In the randomly selected rats (n = 6) for each group, the development of cirrhosis was histologically confirmed by the presence of fibrotic scar tissues and abnormal nodules, indicative of the excessive deposition of extracellular matrix, primarily consisting of type I collagen (see [17] for details) Additionally, the serum aspartate aminotransferase (AST) and alanine aminotransferase (ALT) levels were determined. The severity of cirrhosis was routinely assessed through visual examination of liver attributes such as color, shape, and surface characteristics (Figure S1) as well as the presence of ascites in the abdominal cavity.

### 2.3. Measurement of Arterial Blood Pressure, Heart Rate, and Portal Pressure

Arterial blood pressure was measured as previously described [17]. Briefly, sham-operated and cirrhotic rats were anesthetized with urethane (0.75 g/kg in 0.9% saline, intraperitoneally). The right femoral artery was cannulated with a PE50 catheter to record the blood pressure and heart rate. To measure portal pressure, a median laparotomy was performed and the hepatic hilum was exposed as previously described [20]. The portal vein was then cannulated with a PE50 catheter through the distal superior mesenteric vein. All catheters were connected to strain gauge pressure transducers. Hemodynamic parameters were recorded using a PowerLab data acquisition system equipped with LabChart v7 software (AD Instruments, Colorado Springs, CO, USA).

### 2.4. Power Spectral Analysis of HRV

ECGs were recorded in rats anesthetized with 2% isoflurane in oxygen, following previously outlined protocols [21]. Three needle electrodes were subcutaneously implanted in the limbs to facilitate the recordings. These electrodes were connected to a Bio amplifier (AD Instruments), and signals were digitized using a PowerLab system (AD Instruments) [22]. ECG data were digitized at 2 kHz and analyzed using Lab Chart v7 software (AD Instruments). Animals were allowed to stabilize for at least 30 min before recordings commenced. The power spectral densities of various frequency components were calculated from R-R interval variability using the fast Fourier transform algorithm. Low frequency (LF) and high frequency (HF) components were analyzed in spectra derived from short-term recordings of 2 min. LF and HF powers were defined as the area under the curve in the frequency ranges of 0.04–1.0 Hz and 1.0–3.0 Hz for rats, respectively [23,24]. The LF/HF power ratio was then calculated to assess the cardiac sympathovagal balance.

### 2.5. Dissociation of Single Cardiac Efferent Neurons

Sympathetic SG and parasympathetic ICG neurons were enzymatically dissociated using a modified version of methods previously described [25,26]. Briefly, rats were anesthetized with urethane (0.75 g/kg in 0.9% saline, intraperitoneally) or 2% isoflurane in oxygen. The SG and ICG were excised and immediately placed in cold Hanks’ balanced salt solution (HBSS, pH 7.4). The ganglia were desheathed, sectioned into small fragments, and transferred to a culture tube containing in modified Earle’s balanced salt solution (EBSS, pH 7.4) with 1 mg/mL collagenase type D (Roche Diagnostics, Mannheim, Germany), 0.15 mg/mL trypsin (Worthington Corporation, Harrison, NJ, USA), and 0.1 mg/mL DNase type I (Sigma-Aldrich). This EBSS was supplemented with 3.6 g/L glucose and 10 mM HEPES (Sigma-Aldrich). The culture tube was then placed in a shaking water bath at 100 rpm and 35.5 °C for 60 min. After incubation, the ganglia were mechanically dispersed into single neurons by vigorous shaking. The dissociated neurons were washed twice, resuspended in minimum essential medium containing 10% fetal calf serum and 1% penicillin–streptomycin solution (both from Invitrogen, Carlsbad, CA, USA), and plated onto 35 mm polystyrene culture dishes pre-coated with poly-D-lysine (Sigma-Aldrich). The cultures were maintained in a humidified incubator with 95% air and 5% CO_2_ at 37 °C until further use.

### 2.6. Single-Cell Real-Time PCR Analysis

Single-cell real-time RT-PCR was performed as previously described [27]. Each dissociated neuron was aspirated into a patch pipette using negative pressure and deposited into a 0.2-mL PCR tube containing the following: 1 μL lysis buffer (0.25 M Tris·HCl, 0.275 M KCl, 0.015 M MgCl_2_, 2.5% Nonidet P-40), 0.5 μL RNA guard mix, 2 μL RNase inhibitor (Applied Biosystems, Foster City, CA, USA), and 1.5 μL DEPC-treated water, totaling 5 μL. Tubes were stored at −80 °C until reverse transcription. First-strand cDNA synthesis was conducted using 5 μL of the lysate in each tube with the Sensiscript Reverse Transcriptase Kit (Qiagen, Valencia, CA, USA), according to the manufacturer’s instructions. The synthesized cDNA was subsequently stored at −80 °C until real-time PCR. Primers for real-time PCR, listed in Table 1, included both outer and inner (nested or semi-nested) sets to enhance specificity and sensitivity. Primers were designed to amplify cDNA segments spanning two or more exons to prevent genomic DNA amplification. PCR reactions were conducted in a 25 μL mixture containing 12.5 μL SYBR Green master mix (ABI), 200 nM of each primer in the first round, and 300 nM in the second round. The protocol entailed preheating at 94 °C for 10 min, followed by 30–35 cycles of 94 °C for 15 s, 60 °C for 1–2 min, and 72 °C for 2 min, with a final extension at 72 °C for 5 min on a 7900HT Fast Real-Time PCR System (Applied Biosystems). The β-actin gene was amplified as an internal reference. Gene expression fold changes were analyzed using the comparative 2^−ΔΔCT^ method [28].

### 2.7. Patch-Clamp Recordings

Current-clamp and voltage-clamp recordings were performed under the gramicidin-perforated and whole cell-ruptured configurations of the patch-clamp technique, respectively, employing an EPC-10 amplifier and pulse/pulsefit software v8.50 (HEKA Electronik, Lambrecht, Germany), as previously described [17,29]. For current-clamp recordings, the external solution was a normal physiological salt solution with the following composition (in mM): 135 NaCl, 5.4 KCl, 10 HEPES, 10 glucose, 1.8 CaCl_2_, and 1.0 MgCl_2_6H_2_O, adjusted to pH 7.2 and 318 mOsm/Kg H_2_O. The gramicidin stock solution was prepared at 50 mg/mL in DMSO and diluted to a final concentration of 50 μg/mL in an internal solution containing 120 K-gluconate, 20 KCl, 10 EGTA, and 10 HEPES (pH 7.2, 310 mOsm /Kg H_2_O). For the K^+^ current recordings, the external solution consisted of (in mM): 150 choline-Cl, 5 KCl, 10 HEPES, 1.5 CaCl_2_, 5 MgCl_2_, 0.2 CdCl_2_, and 10 glucose, adjusted to pH 7.4 and 325 mOsm/Kg H_2_O [22]. For the Ca^2+^ current recordings, the external solution contained 145 mM tetraethylammonium methanesulfonate (MS), 10 mM HEPES, 10 mM CaCl_2_, 15 mM glucose, and 0.0003 mM tetrodotoxin, adjusted to 7.4 and 318 mOsm/kg H_2_O [29]. Patch pipettes, fabricated from borosilicate glass capillaries (#8250, King Precision Glass, Inc., Claremont, CA, USA), were filled with specific internal solutions for A-type and delayed rectifier K^+^ currents (140 KCl, 5 EGTA, 1 MgCl_2_, 0.5 CaCl_2_, 10 HEPES, 3 Mg-ATP, 3 Na-GTP, pH 7.3, 310 mOsm/Kg H_2_O) or M-type K^+^ currents (140 KCl, 0.1 BAPTA, 10 HEPES, 4 Mg-ATP, 0.1 Na-GTP, pH 7.4, 310 mOsm/Kg H_2_O) or Ca^2+^ currents (120 N-methyl-D-glucamine-MS, 20 TEA-MS, 20 HCl, 11 EGTA, 1 CaCl_2_, 10 HEPES, 4 MgATP, 0.3 Na_2_GTP, 14 creatine phosphate, pH 7.2, 297 mOsm/Kg H_2_O). Electrodes were coated with Sylgard 184 (Dow Corning, Midland, MI, USA) and fire-polished to achieve a resistance of 2–3 MΩ. Cell membrane capacitance and series resistance were electronically compensated to over 80%. Drugs were applied to neurons via a gravity-fed system using a fused silica capillary tube connected to an array of seven polyethylene tubes. Stock solutions (0.1–10 mM) of CdCl_2_, nimodipine (all from Sigma-Aldrich), and ω-conotoxin GVIA (Alomone Labs, Jerusalem, Israel) were prepared, with nimodipine dissolved in DMSO and others in distilled water. Experiments were conducted at room temperature (22–24 °C).

### 2.8. Data Analysis and Statistics

Electrophysiological data were analyzed using the IGOR data analysis package (v4.0.2.1, WaveMetrics, Lake Oswego, OR, USA) and GraphPad Prism (v8.0.1, GraphPad Software Inc., La Jolla, CA, USA). Data are presented as means ± SEM. Statistical significance was assessed using the Student’ t-test or one-way ANOVA followed by Tukey’s multiple comparison post hoc test. A *p*-value of less than 0.05 was considered statistically significant.

## 3. Results

### 3.1. Evaluation of Blood Pressure, Heart Rate, Portal Pressure, and Myocardial Mass in Normal and Cirrhotic Rats

All animals of the CBDL and TAA groups were confirmed to have cirrhosis through various methods including histology of the liver tissue sections [17], measurement of portal pressure [30], and visual examination of liver attributes such as color, shape, and surface characteristics (Figure S1) as well as the presence of ascites in the abdominal cavity.

The hemodynamic characteristics of normal (sham-operated and sham-saline) and cirrhotic rats are summarized in Table 2. Consistent with prior research [17,31,32], there were no significant differences in the baseline mean arterial pressure (MAP) and heart rate (HR) between normal and cirrhotic rats. Specifically, the MAP averaged 93 ± 4 mmHg in the sham-operated (n = 5) and 86 ± 5 mmHg in the CBDL rats (n = 6) rats, while it was 92 ± 4 mmHg in the sham-saline rats (n = 5) and 87 ± 4 mmHg in the TAA rats (n = 6). HR averaged 336 ± 6 beats/min in the sham-operated rats and 323 ± 12 beats/min in the CBDL rats; in the sham-saline and TAA rats, HR was 341 ± 7 beats/min and 321 ± 11 beats/min, respectively. Consistent with earlier studies [32,33], heart weight significantly increased in the CBDL (*p* < 0.001) and TAA (*p* < 0.05) rats compared to their respective controls. The percentage of heart weight normalized to body weight was 0.25 ± 0.01% in the sham-operated rats and 0.38 ± 0.02% in the CBDL rats, and 0.26 ± 0.01% in the sham-saline rats and 0.34 ± 0.03% in the TAA rats. Portal pressure was significantly higher in the cirrhotic rats (*p* < 0.001), averaging 8.2 ± 0.2 mmHg in the sham-operated rats compared to 15.3 ± 0.4 mmHg in the CBDL rats and 8.3 ± 0.3 mmHg in the sham-saline rats compared to 14.7 ± 0.5 mmHg in the TAA rats. These results suggest the development of portal hypertension, a significant complication associated with cirrhosis, in these experimental models. It is important to note that there were no significant differences between the two control groups in all parameters except body weight. These standards also apply to HRV analysis, electrophysiology, and single-cell RT-PCR studies.

### 3.2. Decrease in HRV in Cirrhotic Rats

**Figure 2:**
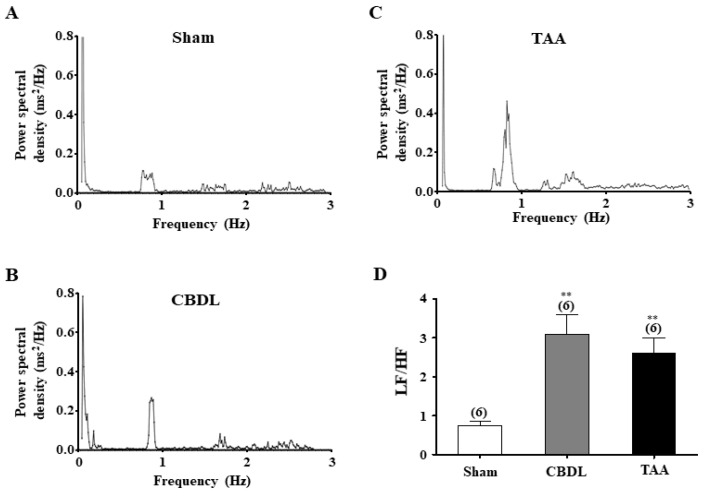
Power spectral analysis of HRV in normal and cirrhotic rats. Representative traces show the power spectral densities (PSDs) of various frequency components calculated from the R-R interval variability using the fast Fourier transform algorithm in sham (**A**), CBDL (**B**), and TAA rats (**C**). (**D**) Summary of the LF/HF power ratio in normal and cirrhotic rats. Total power densities were calculated within the frequency range of 0 to 3 Hz. LF and HF powers were defined as the area under the curve in the frequency ranges of 0.04–1.0 Hz and 1.0–3.0 Hz for rats, respectively. Data are presented as the mean ± SEM. The number of experiments is indicated in parentheses. ** *p* < 0.01 compared with normal rats.

We assessed CAD in cirrhotic rats using the power spectral analysis of HRV. Figure 2A–C depicts the representative power spectra of R-R interval variability for both the normal (sham-control) and cirrhotic rats. In cirrhotic rats, both the total power and high frequency (HF) components were significantly decreased (*p* < 0.05), while the low frequency (LF) components significantly increased (*p* < 0.05) compared to normal rats. Specifically, the average total power, LF, and HF (in ms^2^) were 12.1 ± 1.7, 1.6 ± 0.3, 2.1 ± 0.4 in normal rats (n = 6); 6.3 ± 1.2, 1.8 ± 0.2, 0.6 ± 0.2 in CBDL rats (n = 6); and 6.5 ± 0.7, 1.9 ± 0.2, 0.5 ± 0.2 in TAA rats (n = 6), respectively. Importantly, the LF/HF ratio, indicative of sympathovagal balance, was significantly higher in the CBDL (*p* < 0.01) and TAA (*p* < 0.001) groups compared to the normal rats, as shown in Figure 2. Average LF/HF ratios were 0.8 ± 0.5 for normal rats, 3.1 ± 0.5 for CBDL rats, and 2.6 ± 0.4 for TAA rats. These findings indicate a marked development of autonomic imbalance, characterized by excessive sympathetic activity and diminished parasympathetic activity in cirrhotic rats.

### 3.3. Cirrhosis-Induced Alterations in the Excitability of Cardiac Efferent Neurons

**Figure 3:**
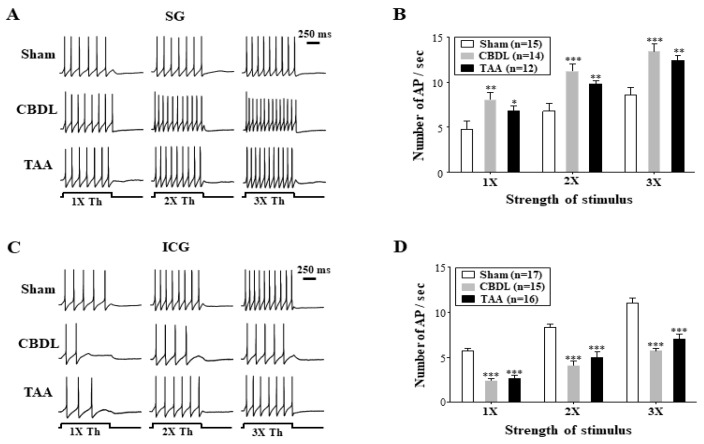
Cirrhosis-induced alterations in the excitability of SG and ICG neurons. Representative traces of AP discharges in response to depolarizing current steps to 1, 2, and 3 times-threshold (1×, 2×, and 3× Th) for 1 s in (**A**) SG, (**C**) ICG neurons from the sham, CBDL, and TAA rats. Each neuron was depolarized from a resting membrane potential between −51 and −56 mV. All recordings were performed under the gramicidin-perforated configuration of the whole-cell current-clamp technique. (**B**,**D**) Summary of the number of spikes per second measured respectively in SG and ICG neurons in the sham, CBDL, and TAA rats. Data are presented as the mean ± SEM. The number of neurons tested is indicated in parentheses. * *p* < 0.05, ** *p* < 0.01, *** *p* < 0.001 compared with the sham-operated rats.

We investigated the impact of cirrhosis-induced autonomic imbalance on the excitability of sympathetic SG and parasympathetic ICG neurons, which innervate the heart [33,34]. Consistent with prior research [22,35,36], SG and ICG neurons in normal rats exhibited regular and sustained action potential (AP) discharge in response to depolarizing current injections (1× threshold) for 1 s under the gramicidin-perforated patch-clamp configuration (Figure 3A,C). In cirrhotic rats, significant changes in neuronal excitability were observed. SG neurons showed increased excitability in both the CBDL and TAA models (*p* < 0.001 and *p* < 0.01, respectively; Figure 3A,B), with firing rates substantially higher than in normal rats. Specifically, normal SG neurons (n = 15) discharged at rates of 4.8 ± 0.7, 6.8 ± 0.8, and 8.6 ± 0.8 AP/s in response to 1×, 2×, and 3× threshold current stimulations, respectively. In contrast, the CBDL rats (n = 14) and TAA rats (n = 12) exhibited higher firing rates of 8.0 ± 0.7, 11.2 ± 0.8, 13.4 ± 0.8 AP/s and 6.8 ± 0.6, 9.8 ± 0.3, 12.4 ± 0.5 AP/s, respectively, across the same stimulation levels. Conversely, the excitability in ICG neurons significantly decreased in these models (*p* < 0.001 for both; Figure 3C,D), with lower firing rates than normal rats.. Specifically, normal ICG neurons (n = 17) discharged at rates of 5.6 ± 0.6, 8.3 ± 0.8, and 11.0 ± 0.8 AP/s in response to 1×, 2×, and 3× threshold current stimulations, respectively. The ICG neurons of the CBDL rats (n = 15) fired at 2.3 ± 0.1, 4 ± 0.3, and 5.7 ± 0.3 AP/s, and those of the TAA rats (n = 16) at 2.7 ± 0.1, 5 ± 0.4, and 7.0 ± 0.4 AP/s, respectively, at the same stimulation levels.

Membrane properties of the SG and ICG neurons in both the normal and cirrhotic rats are summarized in Table 3. Notably, cell capacitance and resting membrane potential (RMP) remained consistent across all groups. To further assess the altered excitability in cardiac efferent neurons of cirrhotic rats, we measured the threshold potentials and current thresholds (rheobase) required to trigger a single AP. The SG and ICG neurons were stimulated with incremental depolarizing currents for 5 ms until a single AP was generated. Results showed a significant decrease in the rheobase in the SG neurons of CBDL (32 ± 4 pA) and TAA (38 ± 3 pA) rats compared to the normal (61 ± 4 pA) rats (*p* < 0.05), indicating heightened neuronal excitability. Concurrently, the threshold potential in SG neurons was significantly lowered in cirrhotic rats (−30 ± 1.3 mV for normal, −38.3 ± 1.6 mV for CBDL, −37.7 ± 1.3 mV for TAA), suggesting easier neuronal activation. Conversely, in the ICG neurons, both the rheobase and threshold potential increased significantly in the cirrhotic rats, indicative of reduced excitability. The rheobase values were 54 ± 4 pA in the normal, 87 ± 4 pA in the CBDL, and 90 ± 4 pA in the TAA rats. Threshold potentials were −31.3 ± 1.3 mV in the normal, −21.7 ± 1.1 mV in the CBDL, and −22 ± 1 mV in the TAA rats. Additionally, changes in AP and afterhyperpolarization (AHP) durations were also noted, with SG neurons showing shorter durations in cirrhotic rats (AP duration: 2.9 ± 0.1 ms in normal, 2.1 ± 0.2 ms in CBDL, 2.2 ± 0.2 ms in TAA; AHP duration: 287 ± 18 ms in normal, 221 ± 15 ms in CBDL, 234 ± 17 ms in TAA), and ICG neurons showing prolonged durations (AP duration: 2.1 ± 0.2 ms in normal, 3.1 ± 0.2 ms in CBDL, 2.9 ± 0.2 ms in TAA; AHP duration: 222 ± 13 ms in normal, 298 ± 18 ms in CBDL, 303 ± 17 ms in TAA). Collectively, these findings indicate that cirrhosis induces opposite changes in the excitability of sympathetic and parasympathetic cardiac efferent neurons, contributing to the autonomic imbalance observed in cirrhotic rats.

### 3.4. Downregulation of A-Type K^+^ Currents in SG Neurons of Cirrhotic Rats

**Figure 4:**
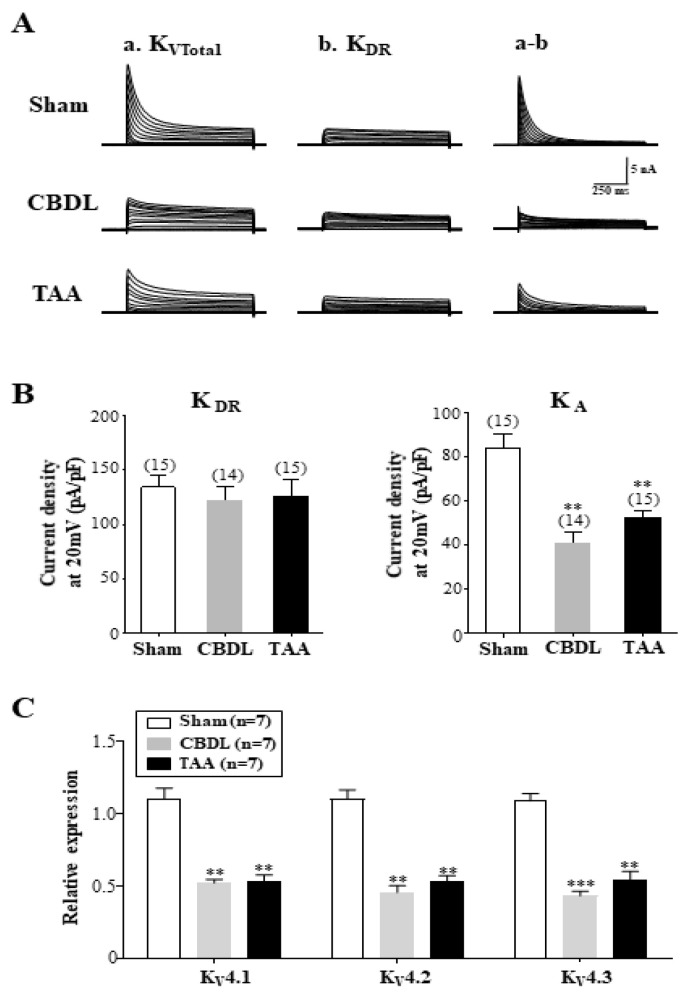
Downregulation of A-type K^+^ currents in the SG neurons of cirrhotic rats. (**A**) Representative traces of the total, delayed rectifier (K_DR_), and A-type (K_A_) K^+^ currents recorded in the SG neurons of the sham-operated, CBDL, and TAA rats. Total outward K^+^ (K_VTotal_) and K_DR_ currents were elicited by 1-s depolarizing pulses ranging from −50 mV to +20 mV, starting from holding potentials of −100 mV and −60 mV, respectively. K_A_ current traces were derived by subtracting the K_DR_ current from the K_VTotal_ currents. (**B**) Summary of the K_DR_ and K_A_ current densities measured at −20 mV in the SG neurons of the sham, CBDL, and TAA rats. (**C**) Summary of the relative expression of the transcripts encoding Kv4.1, Kv4.2, and Kv4.3 in the SG neurons of the sham, CBDL, and TAA rats. Data are presented as the mean ± SEM. The number of neurons tested is indicated in parentheses. ** *p* < 0.01, *** *p* < 0.001 compared with the sham rats.

Unlike baroreceptor neurons [17], voltage-dependent sodium currents remain unaltered in the cardiac efferent neurons of cirrhotic rats (Figure S2). Voltage-dependent K⁺ (K_V_) currents, which are crucial for modulating activation thresholds and spike frequencies, have been extensively studied in sympathetic neurons, which express both transient A-type K⁺ (K_A_) and sustained delayed rectifier K⁺ (K_DR_) currents [37,38,39,40,41]. In this study, we investigated whether K_A_ and K_DR_ currents were altered in the SG neurons of cirrhotic rats. Total outward K^+^ (K_VTotal_) and K_DR_ currents were elicited by 1-s depolarizing pulses ranging from −50 mV to +20 mV, starting from holding potentials of −100 mV and −60 mV, respectively (Figure 4A). K_A_ current traces were isolated by subtracting the K_DR_ current from the K_VTotal_ currents [42]. Our results indicate a significant reduction in the K_A_ currents but not in the K_DR_ currents in the SG neurons from the CBDL and TAA rats compared to the normal controls (*p* < 0.01 for both comparisons). Specifically, the K_A_ currents at +20 mV averaged 84 ± 6 pA/pF in the normal rats (n = 15), 41 ± 5 pA/pF in the CBDL rats (n = 14), and 53 ± 3 pA/pF in the TAA rats (n = 15). The K_DR_ current densities were 134 ± 12 pA/pF, 122 ± 13 pA/pF, and 126 ± 15 pA/pF for the normal, CBDL, and TAA rats, respectively (Figure 4B).

Transcripts encoding Kv4.1, Kv4.2, and Kv4.3, which predominantly underlie K_A_ currents, are expressed in SCG neurons [43,44]. Consistently, single-cell real-time PCR analysis revealed the downregulation of transcripts encoding all K_A_ channel α subunits in the SG neurons of cirrhotic rats (Figure 4C). Normalized to β-actin, the relative expression levels of K_V_ 4.1, K_V_ 4.2, and K_V_ 4.3 were 0.52 ± 0.02, 0.45 ± 0.05, and 0.43 ± 0.03, respectively, in CBDL rats (n = 7), and 0.54 ± 0.04, 0.53 ± 0.04, and 0.55 ± 0.06, respectively, in the TAA rats (n = 7) (Figure 4C). Collectively, these findings suggest that the cirrhosis-induced hyperexcitability of SG neurons may arise from the downregulation of K_A_ channels. Previous research has noted that adult ICG neurons do not express functional K_A_ currents [45], corroborated by our findings, as K_A_ currents were undetectable in the ICG neurons [22]. Additionally, cirrhosis did not alter the K_DR_ currents in the ICG neurons.

### 3.5. Differential Regulation of M-Type K^+^ Channels in Cardiac Efferent Neurons of Cirrhotic Rats

Neuronal M-type K^+^ (K_M_) channels are crucial for regulating the subthreshold electrical excitability and AP frequency in both sympathetic and parasympathetic neurons [46,47,48]. Previous research has shown that the application of XE-991 (3 μM), a selective K_M_ channel blocker, significantly increased the AP frequency in SG and ICG neurons [22]. We further examined the K_M_ current deactivation, induced by a 500 ms test pulse to −60 mV from a holding potential of −30 mV. Figure 4A illustrates characteristic K_M_ current traces from the SG and ICG neurons of the normal, CBDL, and TAA rats. We observed a significant decrease in the K_M_ currents in SG neurons (*p* < 0.001) and an increase in the ICG neurons of cirrhotic rats (*p* < 0.001) (Figure 4B). The average KM current density was 2.2 ± 0.2 pA/pF in normal SG neurons (n = 13), 1.0 ± 0.2 pA/pF in the CBDL rats (n = 14), and 1.2 ± 0.2 pA/pF in the TAA rats (n = 14). In contrast, the ICG neurons showed current densities of 2.7 ± 0.1 pA/pF in the normal rats (n = 12), 4.1 ± 0.3 pA/pF in the CBDL rats (n = 13), and 3.5 ± 0.3 pA/pF in the TAA rats (n = 13).

**Figure 5:**
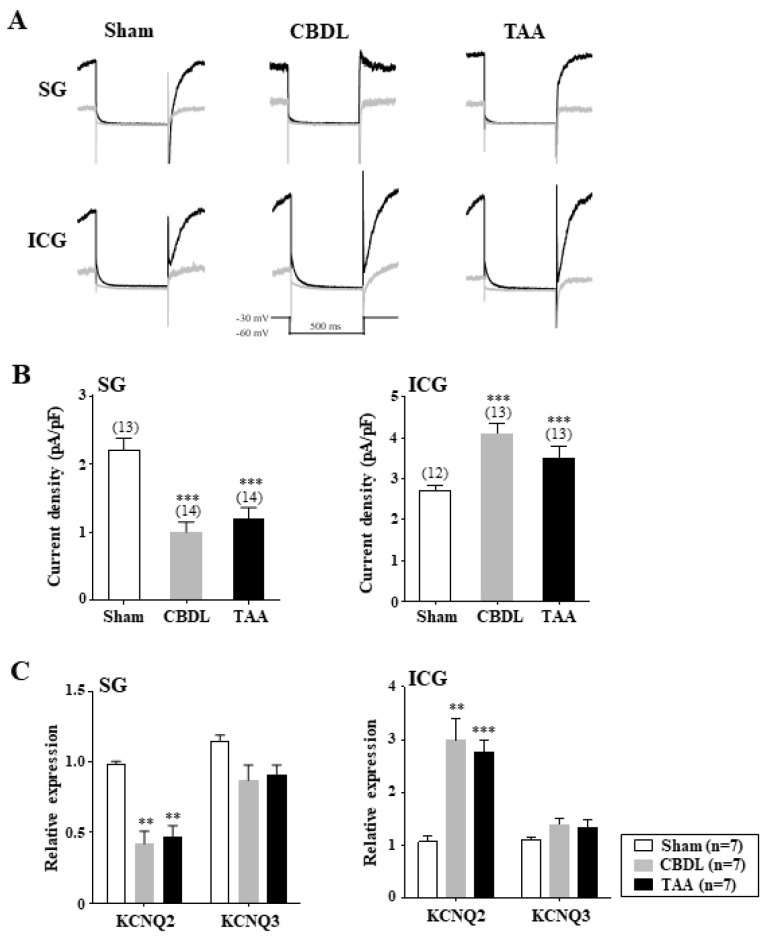
Differential regulation of K_M_ channels in cardiac efferent neurons of cirrhotic rats. (**A**) Representative K_M_ current traces are recorded in the SG and ICG neurons of the sham-operated, CBDL, and TAA rats. K_M_ currents were deactivated by a 500 ms test pulse to −60 mV from a holding potential of −30 mV (inset shows the pulse protocol). (**B**) Summary of the K_M_ current densities in the SG and ICG neurons of the sham, CBDL, and TAA rats. (**C**) Summary of the relative expression of transcripts encoding KCNQ2 and KCNQ3 in the SG and ICG neurons of the sham, CBDL, and TAA rats. Data are presented as the mean ± SEM. The number of neurons tested is indicated in parentheses. ** *p* < 0.01, *** *p* < 0.001 compared with the sham rats.

Native K_M_ channels are comprised of KCNQ2 and KCNQ3 subunits in rat autonomic neurons [49,50]. Single-cell real-time PCR analysis revealed that the transcript encoding KCNQ2 was approximately 2-fold downregulated in the SG neurons and 2.5–3-fold upregulated in the ICG neurons of cirrhotic rats compared to normal rats (Figure 5C). However, the expression of the KCNQ3 transcripts remained unchanged in both neuron types of cirrhotic rats. These findings indicate that cirrhosis differentially regulates K_M_ channels, thereby contributing to distinct alterations in the excitability of cardiac efferent neurons.

### 3.6. Downregulation of L- and N-Type Ca^2+^ Channels in ICG Neurons of Cirrhotic Rats

**Figure 6:**
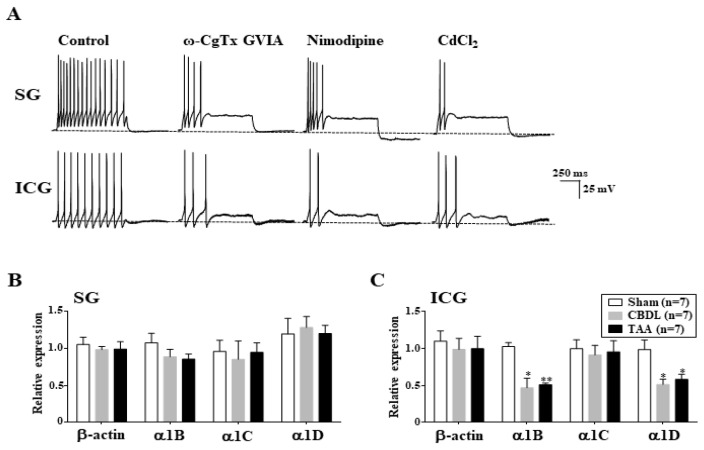
Regulation of AP firing by the inhibition of VDCCs in normal SG and ICG neurons and downregulation of the transcripts encoding N- and L-type Ca^2+^ channel isoforms in the SG and ICG neurons of cirrhotic rats. (**A**) Effects of various VDCCs blockers on AP firing in SG and ICG neurons of normal rats. CdCl_2_ (0.1 mM), a non-specific VDCC blocker, ω-conotoxin GVIA (1 µM), an N-type Ca^2+^ channel blocker, and nimodipine (10 µM), an L-type Ca^2+^ channel blocker were bath applied. Each neuron was depolarized from a resting membrane potential (SG neuron: −52 mV and ICG neuron: −54 mV). All recordings were performed under the gramicidin-perforated configuration of the whole-cell current-clamp technique. (**B**,**C**) Relative expression of the transcripts encoding N-type (α1B) and L-type (α1C and α1D) VDCCs, normalized to the β-actin transcript. Data are presented as the mean ± SEM. The number of neurons tested is indicated in parentheses. * *p* < 0.05, ** *p* < 0.01 compared with the sham rats.

Voltage-dependent Ca^2+^ channels (VDCCs) are crucial in regulating neuronal excitability, influencing pacemaking, AP duration, and AHP, which collectively affect the AP upstroke and AP frequency [51]. Consistent with their roles, the administration of cadmium (0.1 mM), a non-specific VDCC blocker, ω-conotoxin GVIA (1 µM), an N-type Ca^2+^ channel blocker, and nimodipine (10 µM), an L-type Ca^2+^ channel blocker, all resulted in significant reductions in AP frequency in the SG and ICG neurons from adult rats (Figure 6A). These results underscore the roles of specific Ca^2+^ channel subtypes in modulating neuronal excitability under physiological conditions. Further investigation through single-cell real-time PCR analysis revealed a significant decrease in transcripts encoding the α1B (Cav2.2) and α1D (Cav1.3) subunits of VDCCs, specifically in ICG neurons, but not in the SG neurons of the CBDL and TAA rats compared to normal rats (Figure 6B,C). Specifically, the relative expression of α1B and α1D, normalized to the β-actin transcript were 0.47 ± 0.15 and 0.50 ± 0.07, respectively, in the CBDL rats (n = 7), and 0.51 ± 0.02 and 0.58 ± 0.07, respectively, in the TAA rats (n = 7). The expression patterns in the TAA rats were notably similar to those in the CBDL rats.

We recorded the Ca^2+^ currents in the SG and ICG neurons for a comparison between the normal and cirrhotic rats. In the SG neurons of cirrhotic rats, the Ca^2+^ currents remained unaltered across all activation ranges of the current–voltage relation curve (Figure S3). Peak Ca^2^⁺ current densities averaged 60 ± 5 pA/pF (n = 10) in the normal rats, 67 ± 9 pA/pF (n = 9) in the CBDL rats, and 62 ± 6 pA/pF (n = 9) in the TAA rats. The contribution of L-type and N-type Ca^2+^ currents to the total Ca^2+^ currents in SG neurons was similar across the three groups. Specifically, the percent inhibition of Ca^2^⁺ currents following the application of nimodipine (10 µM) was 19 ± 2% (n = 9), 17 ± 3% (n = 9), and 18 ± 2% (n = 9) in the normal, CBDL, and TAA rats, respectively. The ω-conotoxin GVIA (1 µM)-sensitive N-type Ca^2^⁺ currents accounted for 59 ± 4% (n = 10), 63 ± 6% (n = 9), and 62 ± 5% (n = 9) in the normal, CBDL, and TAA rats, respectively.

**Figure 7:**
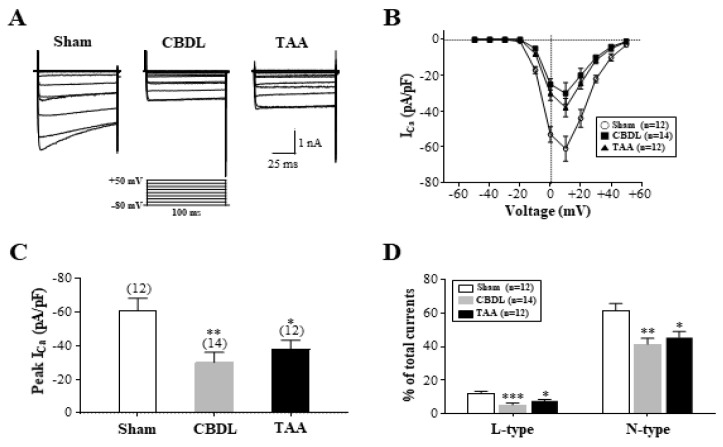
Downregulation of L− and N−type Ca^2+^ channels in the ICG neurons of cirrhotic rats. (**A**) Representative traces of Ca^2+^ currents evoked by depolarizing voltage steps ranging from −60 mV to +50 mV from a holding potential of −80 mV (inset shows the pulse protocol). (**B**) Current–voltage relationships of Ca^2+^ channel currents. (**C**) Summary of the peak Ca^2+^ channel currents measured at +10 mV. (**D**) Summary of the relative contributions (% of total currents) of nifedipine-sensitive L-type and ω-conotoxin GVIA-sensitive N-type currents to the total Ca^2+^ currents in the ICG neurons of the sham, CBDL, and TAA rats. Data are presented as the mean ± SEM. The number of neurons tested is indicated in parentheses. * *p* < 0.05, ** *p* < 0.01, *** *p* < 0.001 compared with the sham rats.

Figure 7A displays the representative Ca^2+^ current traces recorded from the ICG neurons of normal and cirrhotic rats. Unlike in the SG neurons, the Ca^2^⁺ currents were significantly decreased across all activation ranges of the current–voltage relation curve in the ICG neurons of cirrhotic rats (Figure 7B). The peak Ca^2^⁺ current densities averaged 61 ± 7 pA/pF (n = 12) in the normal rats, but were reduced to 30 ± 6 pA/pF (n = 14, *p* < 0.01) in the CBDL rats and 38 ± 5 pA/pF (n = 12, *p* < 0.05) in the TAA rats (Figure 7C). Consistent with single-cell RT-PCR data, the percent inhibition of L-type Ca^2^⁺ currents in the ICG neurons was significantly decreased in the CBDL (6 ± 1%, n = 14, *p* < 0.001) and TAA (8 ± 1%, n = 12, *p* < 0.05) rats compared to normal rats (12 ± 1%, n = 12) (Figure 7D). Similarly, the ω-conotoxin GVIA-sensitive N-type Ca^2^⁺ currents were also reduced, being 42 ± 4% (n = 14, *p* < 0.01) in the CBDL rats and 46 ± 4% (n = 12, *p* < 0.05) in the TAA rats compared to 62 ± 4% (n = 12) in the normal rats (Figure 7D). These findings collectively indicate that N- and L-type Ca^2^⁺ channels are selectively downregulated in the ICG neurons of cirrhotic rats, contributing to their hypoexcitability.

## 4. Discussion

The arterial baroreflex is an essential homeostatic mechanism that regulates cardiovascular functions through a complex neural circuitry. This reflex system comprises an afferent limb with primary and secondary baroreceptor neurons located within the nodose ganglia and the nucleus tractus solitarius (NTS) in the brainstem, respectively. These neurons detect and integrate changes in blood pressure and heart rate. Additionally, the circuit includes central sympathetic and parasympathetic motor pathways in the brainstem and spinal cord as well as an efferent limb composed of sympathetic and parasympathetic postganglionic neurons such as SG and ICG neurons. Functional alterations in these neuronal components at various sites within the baroreflex circuitry may contribute to the cellular mechanisms underlying cirrhosis-induced CAD. Initial research on the effects of cirrhosis on neuronal function has shown an increased expression of c-Fos, a neuronal activation marker, in the cardiovascular centers including the NTS and RVLM, potentially enhancing the sympathetic outflow to spinal preganglionic neurons [16,52]. This observation implicates CNS dysfunction in the hyperdynamic circulation syndrome observed in cirrhotic patients [52] and suggests a potential impairment of the arterial baroreflex. Furthermore, our research has documented a significant reduction in the excitability of primary baroreceptor neurons in cirrhotic rats, indicating baroreflex dysfunction [17]. This study further explored whether cirrhosis affects the function of postganglionic cardiac efferent neurons, yielding two major findings. First, cirrhosis-induced CAD is associated with opposing changes in excitability between sympathetic and parasympathetic neurons innervating the heart. Second, these changes in excitability are due to differential regulation of voltage-dependent K^+^ (A- and M-types) and Ca^2+^ (N- and L-types) channels at both the transcript and functional levels, highlighting a complex mechanism of autonomic dysregulation in cirrhosis.

Previous studies have utilized BRS measurements to evaluate the development of CAD in animal models of various diseases including cirrhosis [17], traumatic brain injury (TBI) [22], diabetes [53], and myocardial infarction [54]. However, clinical investigations involving patients with cirrhosis have predominantly employed the power spectral analysis of HRV to assess CAD development, with a particular emphasis on sympathovagal imbalance [9,55]. In the frequency domain analysis of HRV, the HF component is primarily indicative of parasympathetic activity, whereas the LF component mainly reflects sympathetic control [56]. Consequently, the LF/HF ratio is an indicator of the balance between sympathetic and parasympathetic influences on the heart, a metric used in both rat [23] and human studies [57,58]. Our observations in biliary and nonbiliary cirrhotic rats demonstrated significant reductions in BRS [17] and HRV, as illustrated in Figure 1, indicating an impairment of the arterial baroreflex. Notably, an increase in the LF/HF ratio underscores a pronounced autonomic imbalance characterized by heightened sympathetic activity and reduced parasympathetic influence in cirrhotic rats, aligning with previous clinical observations [55].

Previous research has demonstrated that the firing patterns of SG neurons and ICG neurons in adult rats typically exhibit at least two distinct types of firing spikes: tonic and phasic [59,60]. However, our findings revealed that both the SG and ICG neurons in adult rats were characterized exclusively by non-adapting tonic firing action potentials, consistent with more recent investigations [22,35,36]. The reason for this discrepancy in firing patterns remains unclear. Our electrophysiological studies showed increased excitability in sympathetic SG neurons, while excitability in parasympathetic ICG neurons was decreased in cirrhotic rats, corroborating the findings of the HRV spectral analysis. Additionally, spike discharge plasticity in the SG and ICG neurons of cirrhotic rats is linked to substantial changes in active membrane properties. These include alterations in the AP threshold (rheobase), AP duration, and AHP duration, which were modified in opposite directions between these cardiac efferent neurons.

K_V_ channels are essential determinants of neuronal excitability, and variations in K_V_ channel expression contribute to differences in AP waveform and repetitive firing patterns [61,62]. In line with observations in other sympathetic neurons [38,39,43,44], rat SG neurons predominantly express K_A_ and K_DR_ channels, which are distinguished by their gating kinetics and time- and voltage-dependent properties [22]. Typically, K_DR_ channels modulate AP duration by regulating repolarization in tonic sympathetic neurons [63]. K_A_ channels, on the other hand, recover from inactivation around the peak of the AHP and become active during its decay phase. This activation at the subthreshold membrane potential generates outward currents that critically regulate spike frequency by setting the AP threshold [38,43,44]. Interestingly, in cirrhotic rats, K_A_ currents, but not K_DR_ currents, were selectively reduced in the SG neurons. A previous study demonstrated that the minimum injected current required for AP generation (i.e., rheobase) significantly decreases when functional K_V_4 channels are eliminated in central neurons [64]. Consistent with this, reductions in K_A_ currents have been shown to increase the firing rates in primary afferent neurons in various pain models [41]. Similarly, in the SG neurons of cirrhotic rats, a decrease in K_A_ currents is linked to changes in rheobase, potentially increasing the spike frequency—a pattern also observed in the SG neurons of TBI rat models [22]. We previously hypothesized that Kv4 α subunits (K_V_4.1, K_V_4.2, and K_V_4.3) primarily underlie the K_A_ currents in rat SG neurons, although the exact molecular correlates of these currents remain undefined [22]. Parallel to observations in TBI rat models [22], the downregulation of transcripts encoding these Kv4 isoforms may contribute to the heightened excitability of SG neurons in cirrhotic rats.

To form A-type K^+^ channels in neurons, the Kv4 α subunit assembles with K^+^ channel interacting proteins (KChIPs), which promotes surface expression or stabilizes the presence of Kv4 channel proteins at the surface [65]. Previous studies have demonstrated that auxiliary proteins including KCHIPs are differentially localized in the brain and primary sensory afferent neurons [66,67]. Notably, K_A_ currents, present in SG neurons, were not detected in the ICG neurons of adult rats, a finding consistent with observations in neonatal ICG neurons [45]. The basis for this tissue-specific difference in K_A_ current expression in autonomic neurons remains unclear. In our investigations, we noted that ICG neurons from adult rats express transcripts encoding Kv4 channels at high levels, but those encoding KChIPs (specifically KChIP2 and KChIP4 isoforms) are present at very low levels (Lee and Jeong, unpublished observation). Consequently, it would be informative to test the effects of knocking out or knocking down different auxiliary proteins in SG neurons as well as the effects of the heterologous expression of different auxiliary proteins in ICG neurons on A-type currents and cellular excitability.

K_M_ (K_V_7) channels, a subfamily of K_V_ channels, generate slowly activating and deactivating K^+^ currents in response to membrane depolarization, playing a crucial role in regulating subthreshold electrical excitability and spike frequency in both sympathetic and parasympathetic neurons [22,46,47,48]. Contrary to previous suggestions that tonic firing sympathetic neurons do not express K_M_ currents [47], our findings demonstrated hyperexcitability in tonic firing SG and ICG neurons upon exposure to XE-991, a selective K_M_ channel blocker [22]. In autonomic neurons, KCNQ2 (K_V_7.2) and KCNQ3 (K_V_7.3) channel subunits primarily assemble to constitute native K_M_ currents [49,50]. Cirrhosis selectively modulates KCNQ2 transcript expression, potentially affecting K_M_ currents in both SG and ICG neurons—a modulation also noted in cardiac efferent neurons of TBI rat models [22]. A reduction in K_M_ currents, alongside decreased K_A_ currents, likely contributes to the lowered AP threshold and rheobase, increasing spike firing in SG neurons, a finding paralleled in primary sensory neurons [68]. Conversely, an increase in K_M_ currents in ICG neurons of cirrhotic rats may elevate the threshold and rheobase for AP generation, thereby decreasing spike firing. The mechanisms underlying the selective and differential modulation of KCNQ2 transcript expression and activity in cardiac efferent neurons remain to be elucidated (see below). However, the selective modulation of the KCNQ2 transcript expression in the cardiac efferent neurons of cirrhotic rats is consistent with the notion that the regulation of KCNQ2 expression is essential for determining K_M_ currents in sympathetic neurons [49] and central neurons [69].

The altered excitability of cardiac efferent neurons in cirrhotic rats cannot be solely attributed to the modulation of K_V_ channels (i.e., K_A_ and K_M_). Significant evidence highlights the role of changes in VDCCs. Specifically, reductions in N-type Ca^2^⁺ channel expression and currents, as observed in pathological conditions such as type 2 diabetes [35] and heart failure [36], reduce excitability in ICG neurons. A recent study has demonstrated that targeting Ca_V_2.2 expression in atrioventricular ICG neurons with ShRNA induced ventricular arrhythmia in the experimental rats [70]. These reductions in the N-type Ca^2+^ channel expression prolong the AP duration, leading to decreased spike frequency. Pharmacological evidence demonstrates that SG neurons express the L-, N-, and P/Q-type Ca^2+^ channels [71], while ICG neurons express the L-, N-, Q, and R-type Ca^2+^ channels [72]. The administration of Ca^2+^ channel blockers including nimodipine (L-type), ω-conotoxin GVIA (N-type), and CdCl_2_ (non-specific) consistently reduces the spike frequency in both SG and ICG neurons, emphasizing the crucial role of VDCCs in modulating neuronal excitability. Furthermore, cirrhosis selectively downregulates transcripts encoding α1B (Cav2.2) and α1D (Cav1.3) in ICG neurons. This downregulation corresponds with reductions in pharmacologically dissected N- and L-type Ca^2^⁺ currents, leading to reduced peak Ca^2^⁺ current density in ICG neurons. While stable Ca_V_2.2 mRNA and protein levels typically result in increased N-type Ca^2^⁺ currents in the SG neurons of rats with heart failure, pointing to possible post-translational modifications [36], similar changes in VDCC expression and currents were not observed in the SG neurons of cirrhotic rats, thus dissociating them from cellular hyperexcitability. It remains unclear why Ca^2^⁺ channel expression was not affected in the SG neurons of cirrhotic rats (Figure S3).

The mechanisms underlying the hypoexcitability of ICG neurons due to reductions in VDCCs remain elusive. Patch-clamp studies suggest that Ca^2+^-activated K^+^ currents constitute approximately 35% of the total outward K^+^ current in neonatal ICG neurons, potentially contributing to the generation of AHP [45]. Our subsequent experiments indicated that iberiotoxin, a blocker of large conductance Ca^2^⁺-activated K⁺ channels (BK_Ca_), reduced the spike frequency, whereas apamin, a blocker of small conductance Ca^2^⁺-activated K⁺ channels (SK_Ca_), increased it in both the SG and ICG neurons (Figure S4A). These results suggest that BK_Ca_ currents support repolarization, while SK_Ca_ currents modify AHP amplitude and duration in these neurons. Additionally, cirrhosis did not alter the transcriptional expression of BKα and β subunits or SK2 and SK3 isoforms (Figure S4B,C), implying that the hypoexcitability induced by reduced Ca^2^⁺ currents may be linked to altered BK_Ca_ channel activity rather than changes in gene expression in ICG neurons. Additionally, reductions in Ca^2^⁺ currents did not seem to affect AHP amplitudes or were associated with prolonged AHP duration in the ICG neurons of cirrhotic rats. Further investigation could determine whether KCNQ channels, alongside SK and HCN channels, mediate the medium AHP in both SG and ICG neurons, as observed in CA1 hippocampal neurons [73]. Previous research has demonstrated that cirrhosis significantly decreases sodium currents by downregulating the transcripts and proteins of Na_V_1.7, Na_V_1.8, and Na_V_1.9 in A- and C-type aortic baroreceptor neurons [17]. However, Nav currents in SG and ICG neurons remain unaffected under cirrhotic conditions (Figure S2), highlighting the selective vulnerability of certain neuronal types to cirrhosis-induced changes in ion channel expression.

As discussed, the mechanisms underlying the cirrhosis-induced alterations in the cellular excitability of cardiac efferent neurons are multifaceted, stemming largely from the tissue-specific regulation of various ion channels. To fully understand these complex alterations, further research is imperative to delineate the signaling pathways that govern both the transcriptional and translational regulation of these ion channels. For instance, in primary afferent neurons, the transcription of Kv4.3 and Kv7.2 (KCNQ2) is known to be inhibited by the repressor element 1-silencing transcription factor (REST), which recruits histone deacetylase 4 to bind gene promoters [74,75,76]. Thus, it is crucial to explore whether REST is upregulated in SG neurons under cirrhotic conditions, a change that could significantly affect their excitability and functional outcomes.

As demonstrated in Table 2, hepatic fibrosis induced by CBDL and TAA leads to portal hypertension, primarily by increasing intrahepatic resistance, followed by splanchnic vasodilation and the onset of hyperdynamic circulatory syndrome [77]. In preliminary experiments, we observed significant reductions in both BRS and HRV, indicating impairments in the arterial baroreflex and autonomic balance in a rat model of portal hypertension. Patch-clamp recordings showed differential excitability in autonomic neurons: the SG neurons displayed increased excitability, whereas the ICG neurons were less excitable in portal hypertensive rats, mirroring the findings in cirrhotic rats. These observations suggest a strong link between the development of portal hypertension and cirrhosis-induced autonomic dysfunction. Various circulatory agents including vasopressors such as angiotensin II, endothelin-1, and vasopressin and vasodilators such as nitric oxide, endocannabinoid, and substance P are implicated in cirrhosis-induced portal hypertension and splanchnic vasodilation, respectively [77,78]. Notably, clinical studies have linked arterial baroreflex impairment to enhanced activity of the renin-angiotensin II-aldosterone system in cirrhotic patients [8]. Furthermore, angiotensin II is known to generate superoxide through NADPH oxidase activation and mitochondrial dysfunction, contributing to oxidative stress [79,80]. Previous research suggests that the angiotensin II-NADPH-oxidase-superoxide pathway mediates arterial baroreflex impairment and modulates cellular excitability and ion channel expression in aortic baroreceptor neurons in conditions such as diabetes [27,81] and heart failure [80,82]. Investigating this pathway’s role in autonomic imbalance and altered cellular excitability in the SG and ICG neurons of cirrhotic rats could provide critical insights into the underlying mechanisms of cirrhosis-induced autonomic dysfunction

Emerging research paradigms highlight the physiological and pathophysiological roles of satellite glial cells (SGCs), which closely envelope principal neurons within autonomic ganglia, forming functional units [83,84]. For instance, the ablation of SGCs in mice results in increased sympathetic activity, partly due to the loss of Kir4.1, a glia-specific inwardly rectifying K⁺ channel critical for removing extracellular K⁺ from the vicinity of sympathetic neurons [85]. In the SG of cirrhotic rats, our preliminary experiments indicated differential regulation of Kir4.1 and S100B, a Ca^2^⁺ binding protein. These are sympathetic SGC-specific markers [86] potentially linked to increased neuronal excitability, Furthermore, these pathological conditions also affect the expression of neurotrophic factors such as GDNF and BDNF released from SGCs, influencing neuronal K⁺ currents [87,88]. Consequently, future research should also explore the pathological roles of SGCs in cirrhosis-induced CAD.

In conclusion, our study provides evidence that cirrhosis in rats induces an imbalance between cardiac sympathetic and parasympathetic neuronal activities through differential regulation of the K⁺ and Ca^2^⁺ channels at both the transcript and functional levels. This dysregulation may offer insights into the mechanisms by which cirrhosis causes CAD. Further investigations are necessary to delineate the roles of CNS neurons within the arterial baroreflex circuit and their interactions with peripheral autonomic responses in cirrhosis-induced CAD.

## Figures and Tables

**Table 1:** Single cell real-time PCR primer sequences.

Target Genes	Accession No.	Sequences (5’-3’)	Size (bp)
β-actin	NM031144.2	Fex: ACCAGTTCGCCATGGATGAC	389
		Rex: GGTCTCAAACATGATCTGGG	
		Fin: ATGGTGGGTATGGGTCAGAA	119
		Rin: ACCAACTGGGACGATATGGA	
K_V_4.1	NM001105748.1	Fex: CCGTATATACCACCAGAACC	
		Rex: GGAAGGTTGACTCTCATCTG	610
		Fin: TTGCCAACTCTACTGCGTC	
		Rin: TTGGCATTGAGGCTTGAGC	109
K_V_4.2	NM031730.2	Fex: GGTGATGACAGACAATGAGG	
		Rex: CACAAACTCATGGTTCGTGG	652
		Fin: ACAAACGAAGGGCACAGAAG	
		Rin: AGTTGGTTGCTCAGTAACCC	109
K_V_4.3	NM031739.3	Fex: CATCATCATCTTTGCCACTG	
		Rex: ATTAAGGCTGGAGCGACTAG	733
		Fin: TATTTGGCTCCATCTGCTCC	
		Rin: TTCTTCTGTGCCCTGCGTTT	127
K_V_7.2	NM133322	Fex: ACTGTCCCCATGATCAGCTC	
		Rex: TCTGATGCTGACTTTGAGGC	471
		Fin: GAGTCTCGATGACAGCCCAA	
		Rin: AGGGAGGCTTGCTTCTTCTG	127
K_V_7.3	NM031597.3	Fex: GCAATGTCCTGGCTACCTCT	
		Rex: TTTTGGCTGGCTGCTGCTTC	613
		Fin: CAGCAAAGAACTCATCACCG	
		Rin: ACAGGGCATCAGCATAGGTC	152
Ca_V_1.2	NM012517.2	Fex: AAGATGACTCCAACGCCACC	
		Rex: GATGATGACGAAGAGCACGAGG	385
		Fin: AAGATGACTCCAACGCCACC	
		Rin: AGAGTAGTCCGTAGGCAATC	127
Ca_V_1.3	NM017298.1	Fex: TGAGACACAGACCAAGCGAAGC	
		Rex: GTTGTCACTGTTGGCTATCTGG	376
		Fin: TCCTGCTGAAGCTCTTCTTG	
		Rin: GCTTCCTCTTTCTGAGCAGT	82
Ca_V_2.2	NM147141.1	Fex: TGGAGGGCTGGACTGACAT	
		Rex: GCGTTCTTGTCCTCCTCTGC	283
		Fin: TGGAGGGCTGGACTGACAT	
		Rin: TTGAGCATGAAGAAGGAGCC	109

base pairs, ex: nested external primer, in: nested internal primers; F: forward, R: reverse.

**Table 2:** Hemodynamic characteristics of normal and cirrhotic rats.

Parameters	Sham-Operated (n = 5)	CBDL (n = 6)	Sham-Saline (n = 5)	TAA (n = 6)
Body weight (g)	343 ± 7	325 ± 9	406 ± 9 ^†^	287 ± 8 ***
Heart/body weight (%)	0.25 ± 0.01	0.38 ± 0.03 ***	0.26 ± 0.01	0.34 ± 0.02 *
MAP (mmHg)	93 ± 4	86 ± 5	92 ± 4	87 ± 4
Heart rate (beat/min)	336 ± 6	323 ± 12	341 ± 7	321 ± 11
Portal pressure (mmHg)	8.2 ± 0.2	15.3 ± 0.4 ***	8.3 ± 0.3	14.7 ± 0.5 ***

MAP: mean arterial pressure. Data are presented as means ± SEM. The number of rats tested is indicated in parentheses. * *p* < 0.05, *** *p* < 0.001, compared between sham-operated (normal control) and CBDL rats and between sham-saline (normal control) and TAA rats. ^†^ *p* < 0.05, compared between the controls.

**Table 3:** Membrane properties of the SG and ICG neurons from normal and cirrhotic rats.

Parameters	SG			ICG		
	Normal (n = 15)	CBDL (n = 14)	TAA (n = 12)	Normal (n = 17)	CBDL (n = 15)	TAA (n = 16)
Capacitance (pA/pF)	43 ± 7	48 ± 4	49 ± 4	45 ± 3	41 ± 3	43 ± 4
RMP (mV)	−56 ± 2	−53 ± 2	−51 ± 2	−53 ± 3	−55 ± 2	−53 ± 2
Rheobase (pA)	61 ± 4	32 ± 4 *	37 ± 3 *	54 ± 4	87 ± 4 *	90 ± 4 *
Threshold (mV)	−30.3 ± 1.3	−38.3 ± 1.6 *	−37.7 ± 1.3 *	−31.3 ± 1.3	−21.7 ± 1.1 *	−22 ± 1 *
AP amplitude (mV)	99 ± 4	100 ± 3	100 ± 3	103 ± 1	104 ± 2	106 ± 2
AP duration (ms) at 0 mV	2.9 ± 0.1	2.1 ± 0.2 *	2.2 ± 0.2 *	2.2 ± 0.1	3.1 ± 0.2 *	2.9 ± 0.2 *
AHP amplitude (mV)	18.5 ± 0.5	17 ± 0.9	17 ± 1	16 ± 0.5	17.3 ± 1	18.2 ± 1
AHP duration (ms)	287 ± 18	221 ± 15 *	234 ± 17 *	222 ± 13	298 ± 18 *	303 ± 17 *

AHP, afterhyperpolarization; AP, action potential; RMP, resting membrane potential. Data are presented as means ± SEM. The number of neurons tested is indicated in parentheses. * *p* < 0.05, compared to normal (sham-operated) group.

## Data Availability

The original contributions presented in the study are included in the article/Supplementary Materials, further inquiries can be directed to the corresponding authors.

## Supplementary Materials

The following supporting information can be downloaded at: https://www.mdpi.com/article/10.3390/biomedicines12081722/s1.
