# Supplementary figures and images for: Cellular and Molecular Mechanisms Underlying Altered Excitability of Cardiac Efferent Neurons in Cirrhotic Rats
Source: Biomedicines. 2024 Aug 1;12(8):1722. doi: 10.3390/biomedicines12081722 (PMC11351538; doi:10.3390/biomedicines12081722)

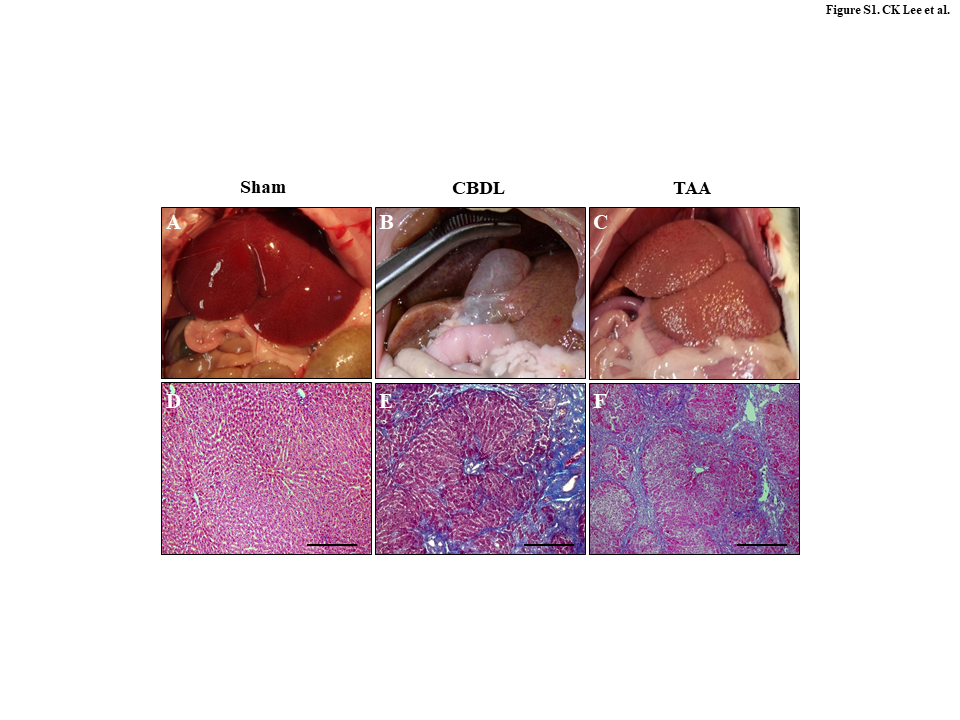

Supplement: Supplementary file 1 [file biomedicines-12-01722-s001.zip › Supplemantary Figures/FIGURE S1.tif]

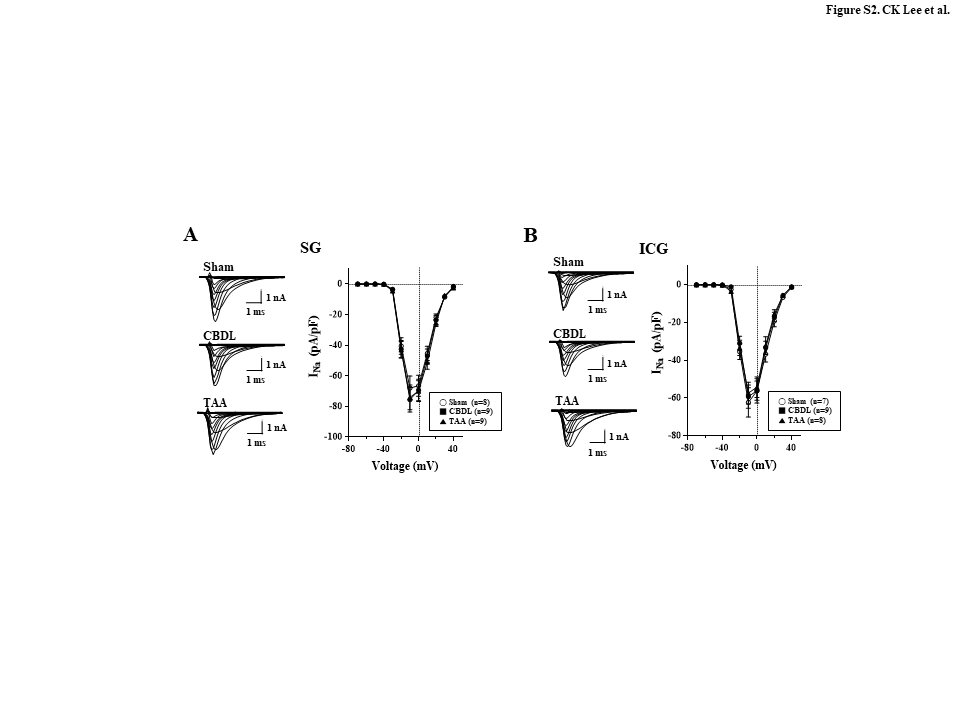

Supplement: Supplementary file 1 [file biomedicines-12-01722-s001.zip › Supplemantary Figures/FIGURE S2.tif]

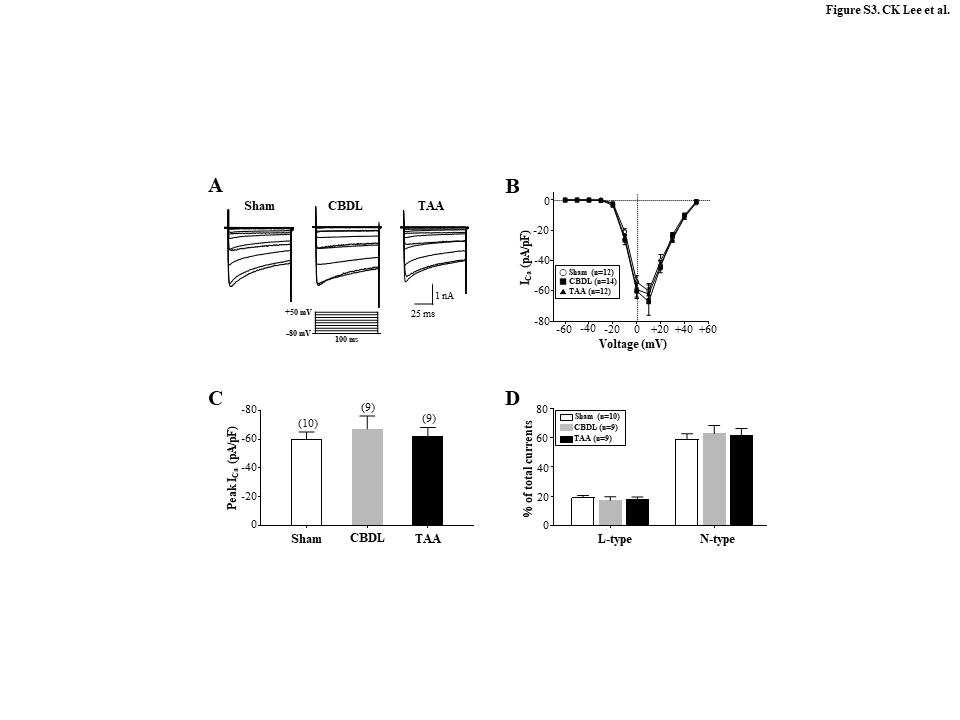

Supplement: Supplementary file 1 [file biomedicines-12-01722-s001.zip › Supplemantary Figures/FIGURE S3.tif]

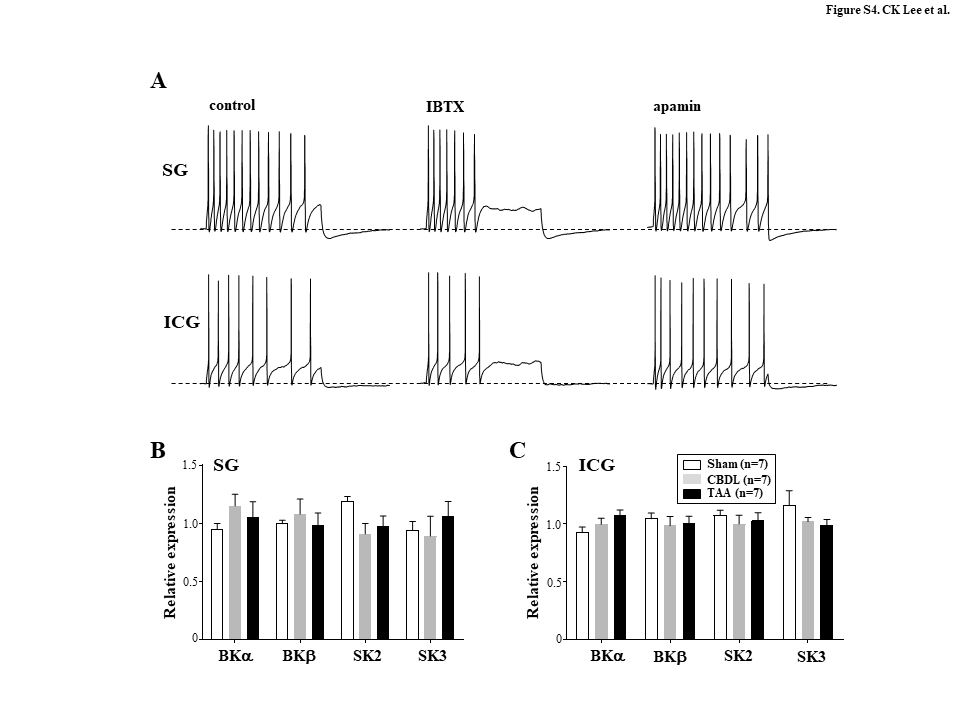

Supplement: Supplementary file 1 [file biomedicines-12-01722-s001.zip › Supplemantary Figures/FIGURE S4.tif]
